# Physiological and Molecular Mechanisms of Light-Induced Greening in Potatoes: A Path to Food Safety

**DOI:** 10.3390/foods14101798

**Published:** 2025-05-19

**Authors:** Xiaohua Zao, Wenli Li, Lixiang Cheng, Bin Yu, Gang Sa

**Affiliations:** 1 State Key Laboratory of Aridland Crop Science, Gansu Agricultural University, Lanzhou 730070, China; 17834455119@163.com (X.Z.); liwl@gsau.edu.cn (W.L.); chenglixiang_0419@163.com (L.C.); 2 College of Agronomy, Gansu Agricultural University, Lanzhou 730070, China

**Keywords:** potato, light condition, greening, chlorophyll, steroidal glycoalkaloids

## Abstract

The potato (*Solanum tuberosum* L.) ranks among the most consumed agricultural products globally and is nutrient-rich. Exposure of potato tubers’ epidermal and subcutaneous tissues to light results in greening and the production of neurotoxic steroidal glycoalkaloids, which significantly reduces tuber quality, increases food safety risks, and leads to rejection by consumers and the processing industry. This review conducts an in-depth analysis of three key aspects: (1) the differences in various light conditions, namely, the disparities in light sources, wavelengths, intensities, and durations; (2) the characteristics of potato cultivars resistant to greening; and (3) the molecular mechanisms of light-induced biosynthesis of chlorophyll and steroidal glycoalkaloids (SGAs). This review is expected to provide technical support for potato food safety measures and a theoretical foundation for the molecular breeding of green-resistant potato varieties.

## 1. Introduction

Potato is one of the most important food crops and vegetables worldwide as a major food source, providing safe and nutritious sustenance [1]. With an annual worldwide yield exceeding 370 million tons, China, with an annual yield of 95 million tons, leads the world in potato production [2]. Potato tubers are the primary edible parts, which are rich in nutrients; therefore, the quality of these tubers plays a crucial role in determining their fresh-eating and suitability for processing. Potato tubers are formed through the transformation and enlargement of underground stolon, and the quality of these tubers greatly varies depending on light conditions, CO_2_ levels, and ambient storage temperatures during post-harvest storage and retail.

However, the greening of tubers caused by light is the primary factor leading to reduced quality, increased storage risk, processing issues, and rejection by both consumers and processing enterprises [3]. It is estimated that 14% to 17% of the U.S. potato crop is lost annually due to tuber greening [4]. At retail, consumer’s willingness to buy potatoes was shown to be negatively affected by greening. For example, in 2009, a survey of 2105 households across 160 locations throughout the U.K. showed that 56% would not purchase potatoes showing evidence of greening, while 40% would throw away uncooked green potatoes [5].Similarly, a survey of six produce managers from major grocery chains in the U.S. reported that green potatoes were routinely discarded due to low consumer purchase rates [6]. The visual appeal of tubers diminished following the light-triggered greening of potato skins, leading to a lower demand from consumers. As underground-modified stems, potato tubers are non-photosynthetic tissues that lack photosynthetic machinery. Potato tubers contain a large number of starch-storing amyloplasts, and upon exposure to light, amyloplasts in the peripheral cell layers develop into chloroplasts that are capable of photosynthesis through the development of photosystems and their associated antenna proteins [7,8,9]. The primary photosynthetic pigments in these proteins are chlorophyll a and b, which are synthesized from the amino acid glutamate through a conserved biosynthesis pathway in the plant kingdom [10]. These pigments reflect predominantly green light, which gives potato tubers a green color [7,8,9,11,12].

**Figure 1:**
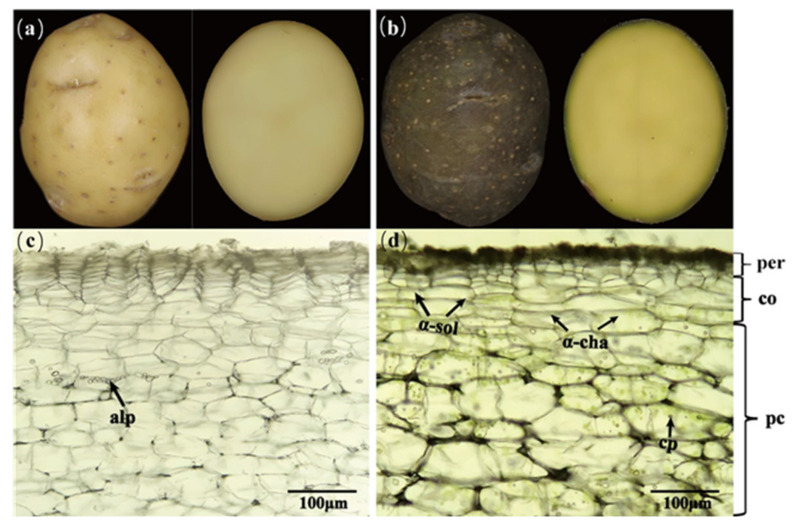
Images of (**a**) potato tuber and (**c**) micrograph of potato tissue beneath the periderm, not exposed to light, (**b**) greening potato tuber, and (**d**) micrograph after 10 days of exposure to 12 μmol m^−2^s^−1^ LED light. Abbreviations: per, periderm; co, cortex; alp, amyloplast; pc, parenchyma cell; cp, chloroplast; α-sol, α-solanine; α-cha, α-chaconine.

Differences in light conditions are closely associated with the photoinduced synthesis of specific glycoalkaloids (GAs) in potatoes, which significantly contributes to consumer aversion to greened potato tubers. The main components of total glycoalkaloids (TGAs) are the trisaccharides α-solanine and α-chaconine, which together comprise approximately 95% of its composition and share the same aglycone, solanidine (steroidal glycoalkaloids, SGAs) [3]. Previous research has indicated that the synthesis and accumulation pathways of steroidal glycoalkaloids (SGAs) in tubers are not linked to the production of photosynthetic pigments associated with tuber greening, but rather represent two distinct metabolic processes [13,14,15,16]. Increased levels of SGAs in potato tubers can lead to a bitter flavor and pungent taste. Furthermore, SGAs remain stable and resistant to degradation during various food processing methods [17,18,19]. Thus, although these two mechanisms function independently via distinct metabolic pathways, they are nevertheless closely associated with visual perception (Figure 1) [14,15].

Greening tubers are regarded as lower quality than their non-greening counterparts and are deemed unsuitable for human consumption due to the associated increase in SGA levels, leading to reduced marketability. There have been over 2000 reported cases of human glycoalkaloid poisoning associated with potato consumption, with fatalities documented in some instances. Additionally, reports indicate that livestock have suffered losses due to the ingestion of potatoes or potato by-products containing glycoalkaloids [20]. As a result, SGAs are now considered among the most toxic substances in the human diet. SGAs remain stable and are not degraded during food processing methods, including boiling, baking, frying, or drying, even at elevated temperatures [19,21].

However, Mensinga et al. demonstrated that the overall SGA levels in tubers do not pose a significant health risk; the half-lives of α-solanine and α-chaconine are 21 and 44 h, respectively. Furthermore, the accumulation of SGAs through daily consumption may present an increased health risk to humans [22]. Consequently, the reduction in potato quality due to greening significantly affects consumer perception and leads to financial losses, thereby influencing consumer purchasing decisions and necessitating a reduction in greening in retail potato sales [3,23].

Previous studies have demonstrated that chlorophyll production and the accumulation of harmful secondary metabolites, such as SGAs, are distinct metabolic processes. However, light conditions, as a critical environmental factor, simultaneously influence both chlorophyll production and SGA levels [3,8,9]. This review offers comprehensive analyses of the physiological changes and molecular mechanisms responsible for tuber greening under diverse lighting conditions. Highlighting the effects of light sources, wavelengths, intensity, duration, and cultivars, the objective is to deepen the understanding of light’s influence on tuber greening (Table 1). Mitigating the risks of tuber greening can be accomplished by adjusting lighting conditions in storage and retail environments, as well as by selecting varieties that are resistant to greening. This approach mitigates the adverse effects of light-induced tuber greening on both food safety and economic losses. By combining the analysis of previous studies on light-induced chlorophyll synthesis and harmful SGA accumulation in tubers—considered distinct processes—we can provide new insights for future research. The concurrent production of chlorophyll and accumulation of SGAs during light-induced tuber greening may be regulated by light signals, highlighting the need for further investigation into the mechanisms governing light signal regulation.

## 2. The Effects of Different Light Conditions on Chlorophyll and SGA Levels in Potatoes

Freshly harvested potatoes are typically displayed indoors under artificial lighting on shelves to attract buyers. However, prolonged light exposure may cause potato skins to turn green, potentially compromising the overall quality of the potatoes. This greening is toxic and generally deters consumer interest [43]. Different artificial lighting conditions can affect the sustainability and durability of fresh potatoes.

### 2.1. Light Sources

Şengül conducted experiments on the storage of two varieties of potatoes, Marfona and Granola, under various light conditions, including normal light storage (NLS), dark storage (NSD), retail refrigerator light (RL), and retail refrigerator dark (RD). The study revealed that under NLS and RL conditions, SGA levels in Marfona and Granola were measured at 17.98 mg/kg and 4.97 mg/kg fresh weight (FW), and 32.76 mg/kg FW and 17.73 mg/kg FW, respectively. These levels were notably higher than those observed under dark storage, indicating that light exposure during storage significantly influences SGA accumulation in potato tubers [40].

Conner et al. reported that after 20 days of incandescent light exposure, SGA content in “Sunburned” tubers (0.0108%) was 2–6 times higher than in filtered light-treated tubers [27]. Salunkhe observed that fluorescent light increased SGAs in Burbank tubers to 7.4 mg/100 g FW, with synthesis rates 3–4 times higher than in darkness, indicating that both incandescent and fluorescent light enhance SGAs accumulation [43]. Machado found SGA levels under sunlight/fluorescent light were 4–6 times higher than in the dark, with fluorescent light inducing more SGAs than sunlight after 14 days [28], likely due to its concentrated blue/red wavelengths favoring toxin biosynthesis, whereas sunlight’s broad spectrum (including UV/far-red) may modulate/inhibit specific pathways. Percival showed that fluorescent light increased SGAs in Pentland Hawk (25.41 mg/100 g FW) and Kerrs Pink (22.49 mg/100 g FW) versus dark storage, confirming artificial light’s higher efficiency in SGA induction due to spectral simplicity [29]. Most experiments controlled temperature (20–25 °C) and humidity (60–70%), isolating light as the primary variable [28,29,43]. However, variable retail environments (15–25 °C, 50–80% humidity) may interact with light to affect greening, warranting future studies on these environmental interactions to optimize storage or display protocols. In 1994, researchers studied the effects of high-pressure mercury, sodium, and fluorescent lighting on SGA production in both dormant and germinating tubers. The results showed that sodium and fluorescent lamps effectively promote SGA synthesis, while high-pressure mercury lighting has a relatively weak effect [30]. By 1999, Percival had determined the chlorophyll and SGA content in tubers of three varieties: Desiree, King Edward, and Kerrs Pink. Tubers irradiated with fluorescent, high-pressure sodium, high-pressure mercury, and low-pressure mercury lamps showed that sodium and fluorescent lamps led to greater glycoalkaloid and chlorophyll accumulation compared to high-pressure and low-pressure mercury lamps. Additionally, chlorophyll synthesis in tubers treated with fluorescent lamps was significantly higher than in those treated with sodium lamps, while sodium lamps showed the highest rate of SGA accumulation [24]. Extensive research has shown that different light sources significantly affect the accumulation of SGAs and chlorophyll in potato tubers, with fluorescent lamps being optimal for chlorophyll accumulation and sodium lamps most effective for SGA accumulation.

**Figure 2:**
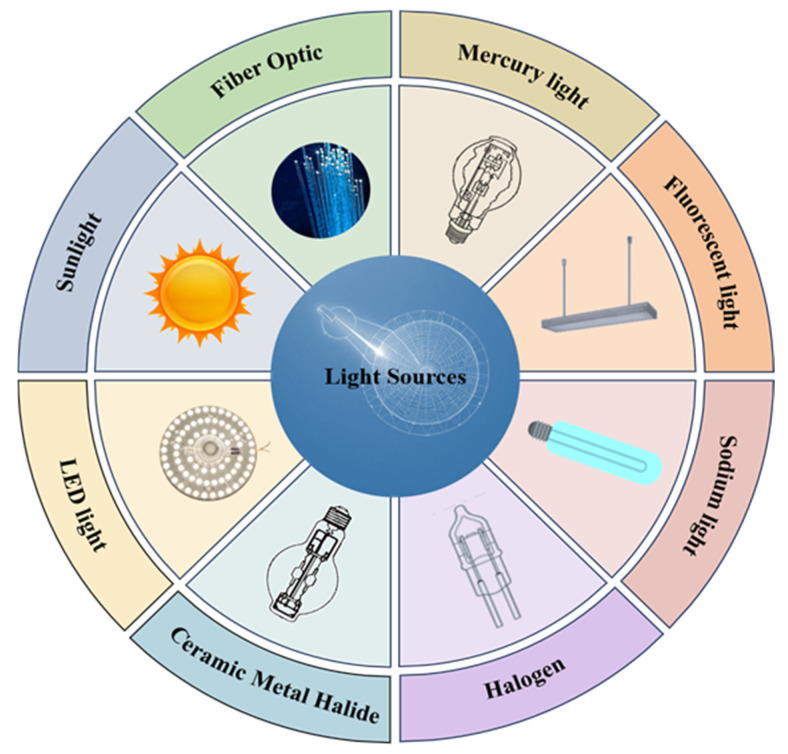
This illustration summarizes the light sources that may cause greening in potato tubers.

Olsen studied the effect of different light sources on the exposed potato tubers of retail shelves. The study investigated the effects of various light sources in illuminated retail settings on chlorophyll and SGA levels of potato tubers. In two experiments, ‘Russet Burbank’ potato tubers were exposed to fiber optic, ceramic metal halide, fluorescent, and filtered fluorescent lighting in the first experiment, and to fiber optic, halogen, and fluorescent lighting in the second experiment. The highest chlorophyll content of 782.4 mg/g FW was observed under ceramic metal halide light. Similar levels were found under fiber optic (543.6 mg/g FW), fluorescent (602.0 mg/g FW), and filtered fluorescent (652.1 mg/g FW) light sources, with no significant differences. Thus, ceramic metal halide lighting exhibited significantly greater chlorophyll levels than fiber optic and fluorescent lighting. The chlorophyll levels were 694.4 mg/g FW under halogen and 628.9 mg/g FW under fluorescent illumination, surpassing the 521.8 mg/g FW found under fiber optic lighting. In both experimental groups, SGA levels remained largely unchanged regardless of the light source. A review of prior studies showed that ceramic metal halide, halogen, filtered fluorescent lamps, fluorescent lamps, and fiber optics were the primary sources of chlorophyll buildup in tubers. The order of light sources resulting in the greatest accumulation of SGAs in tubers was as follows: sodium, fluorescent, mercury, and fiber optic (Figure 2) [25].

Further research is required to evaluate how modern lighting fixtures influence tuber greening and SGA synthesis compared to previous lighting options. This guide helps sellers choose optimal lighting solutions to reduce potato tuber greening and extend shelf life.

### 2.2. Light Wavelengths

Selecting the appropriate lighting wavelengths is also crucial for regulating potato tuber greening and managing the production and accumulation of SGAs. Conner’s research on “Sunburned” potato tubers exposed to red, blue, green, orange, yellow, ultraviolet, and infrared light using seven distinct filters at varying wavelengths revealed that the chlorophyll concentrations under red, orange, and yellow light (103 mg/g, 103 mg/g, and 86 mg/g, respectively) were notably higher than those under other light wavelengths. Conversely, the concentration of SGAs when exposed to blue light reached 31 mg/100 g, surpassing other light wavelengths by 50%, suggesting that SGAs accumulated most rapidly under blue light [27]. These findings indicate that modifying the light source wavelength can effectively manage greening and SGA accumulation in potato tubers. In 1940, Larsen’s research showed significant differences in chlorophyll accumulation between White Rose and Burbank potato tubers under yellow, red, green, and blue light. Exposure to red light led to the highest chlorophyll levels in both varieties, although these levels significantly decreased under blue and green light [37].

In 1960, Yamaguchi exposed White Rose tubers to blue, green, yellow, pink, red, and natural light. Chlorophyll levels were measured at 188 μg/100 cm^2^ under blue light and 164 μg/100 cm^2^ under daylight. By contrast, chlorophyll levels under green, yellow, and red light were 23 μg/100 cm^2^, 33 μg/100 cm^2^, and 33 μg/100 cm^2^, respectively, markedly lower than those under blue light and daylight. Therefore, green, yellow, and red light can inhibit potato tuber greening more effectively than blue light and sunlight. While chlorophyll accumulation was most rapid under blue light, it slowed significantly under green or yellow light [31]. The same year, Liljemark conducted two experiments exploring the effects of different light wavelengths on chlorophyll and SGA levels in potatoes. King Edward potatoes exhibited a three-fold increase in chlorophyll levels under red and green light after 10 days, and a five-fold rise under blue light and sunlight, indicating a faster chlorophyll accumulation rate under blue light and sunlight compared to green and red light. In the second experiment, potato tubers (Bintje, King Edward, and Majestic) were exposed to sunlight in shades of blue, green, and red for 20 days. The results showed that King Edward and Majestic potatoes had low chlorophyll production under green light and high chlorophyll levels under red light. Chlorophyll production in tubers followed the sequence: blue light, sunlight, red light, and green light. The study also found that under red light, the highest SGA accumulation was observed in the Marche and King Edward tubers, with no significant differences observed with other light types on SGA accumulation [32].

With advancements in photovoltaic technology, the precision of different wavelengths has increased. In 1985, Petermann and Morris found that chlorophyll accumulation in potato tubers peaked under blue (475 nm) and red (675 nm) light within the visible spectrum (400–700 nm). Conversely, green (525 nm) and yellow (575 nm) light led to the least chlorophyll accumulation. SGA synthesis was most significant under blue (475 nm) light and red (675 nm) [33]. In 2016, Mekapogu discovered that SGA levels peaked under violet light (147%), followed by red light (108%) and white light (100%), with lower levels observed under blue (83%), green (73%), and UV (42%) light [23].

To further validate the effects of different light wavelengths on the effect of potato tuber greening, Tanios exposed three potato varieties (Nicola, Maranc, and Kennebec) to a single light source at seven specific wavelengths (370, 420, 450, 530, 630, 660, and 735 nm) to monitor tuber greening. The results showed that the highest chlorophyll concentration (4.73 ± 0.66 mg/L) was observed at 450 nm (blue light), followed by white light (2.89 ± 0.49 mg/L), violet light (2.75 ± 0.35 mg/L), green light (2.21 ± 0.39 mg/L), red light (1.65 ± 0.22 mg/L), orange light (1.20 ± 0.48 mg/L), and UV-A light (1.14 ± 0.20 mg/L). The minimal chlorophyll level (0.55 ± 0.11 mg/L) was detected under far-red light (735 nm) [34].

In 2020, Okamoto studied King Edward potato tubers treated with different light wavelengths (blue, red, far-red, and white light) [16]. No significant chlorophyll accumulation occurred in tubers under dark and far-red light conditions, possibly due to the light dependence of the chlorophyll synthesis enzyme protochlorophyllide reductase [44]. Chlorophyll content in tubers consistently increased after exposure to white, blue, and red light, with the synthesis rate ranking as white light > blue light > red light [16].

To further validate research on how different light wavelengths affect key synthetic and rate-limiting enzyme gene expression patterns related to the synthesis of chlorophyll and the accumulation of SGAs in potato tubers, Mekapogu analyzed Atlantic and Haryoung potato tubers to examine how different light wavelengths influence the expression of SGA synthesis genes (*StHMG1*, *StPSS1*, *StSGT1*, *StSGT2*, and *StSGT3*). The study revealed significant variations in gene expression under red, white, green, and yellow light. From an industrial processing perspective, the accumulation of SGAs in greened tubers poses a direct risk to processed products such as French fries and potato chips. Greening potatoes are rejected during the pre-processing screening stage due to their high content of SGAs or appearance defects, which leads to a decrease in the utilization of raw materials. For example, in the production of French fries and potato chips, optical sorting equipment removes greening, sprouted, or defective tubers. Typically, these rejected raw materials are disposed of as animal feed or low-value by-products, which can increase processing costs. The elevated SGA levels in greening potatoes may lead to safety compliance issues in processed products like French fries and potato chips. In the United States, for instance, there is a legal limit of 200 mg/kg for solan glycoalkaloids in fresh potatoes. If greening raw materials are used in processing, this may result in excessive toxin levels in the final products, raising food safety concerns [44,45]. In tubers exposed to red and white light, Haryoung potato exhibited significantly elevated transcript levels for crucial SGA synthesis genes, with *SGT1*’s transcript levels surpassing other genes, increasing by 11 and 8 times, respectively. Similarly, transcript levels of all essential genes were markedly reduced under green and yellow light compared to red and white light. After 24 h treatment, positive associations were observed between tuber SGA levels and gene expression linked to SGA accumulation in red and white light environments. Gene expression for SGAs accumulation decreased under green and yellow light, resulting in significantly lower SGA levels than under red and white light [23].

Okamoto’s research focused on the influence of key enzyme genes (*StHEMA1*, *StHEMA2*, *GSA*, glutamate-1-semialdehyde 2,1-aminotransferase, *StCHLH*, and *StGUN4*) on the chlorophyll synthesis process under various light wavelengths. Exposure to white, blue, and red light significantly increased *StHEMA1* gene expression, with no effect observed in darkness or far-red light. Red and white light also increased *GSA* expression levels compared to blue light. *StCHLH* and *StGUN4* expression was notably higher under white, blue, and red light, with *StGUN4* showing a 100-fold increase under red light. However, no significant difference in chlorophyll synthesis gene expression was observed under far-red light across all tubers. The research further explored SGA accumulation in potato tubers exposed to varied light wavelengths. Genes crucial for SGA synthesis (*StHMG1*, *StSQS*, *StCAS1*, *StSSR2*, *StSGT1*, and *StSGT2*) exhibited varying expression levels under white, blue, and red light, with decreased expression under dark and far-red light. The hypothesis suggests that specific genes activated for glycoalkaloid accumulation throughout the SGA biosynthetic process. Transcriptomic data revealed increased expression of 2097, 2417, and 656 genes under white, blue, and red light wavelengths following 24 h of exposure; expression levels of 325, 694, and 56 genes decreased, respectively, with notable changes in genes involved in biosynthetic pathways, particularly those related to photosynthesis gene activation. Genes directly linked to chlorophyll accumulation, such as *StPSY*, *StHEMA1*, *StCHLH*, and *StGUN4*, experienced significant upregulation under all three lighting wavelengths. Research suggests that cryptochrome and photosensitivity may regulate chlorophyll synthesis and the accumulation of SGAs in potato tubers under irradiation with white, blue, and red light [16]. Despite unchanged biosynthetic enzyme gene expression under far-red light, there was an observable reaction to far-red light irradiation [46].

**Figure 3:**
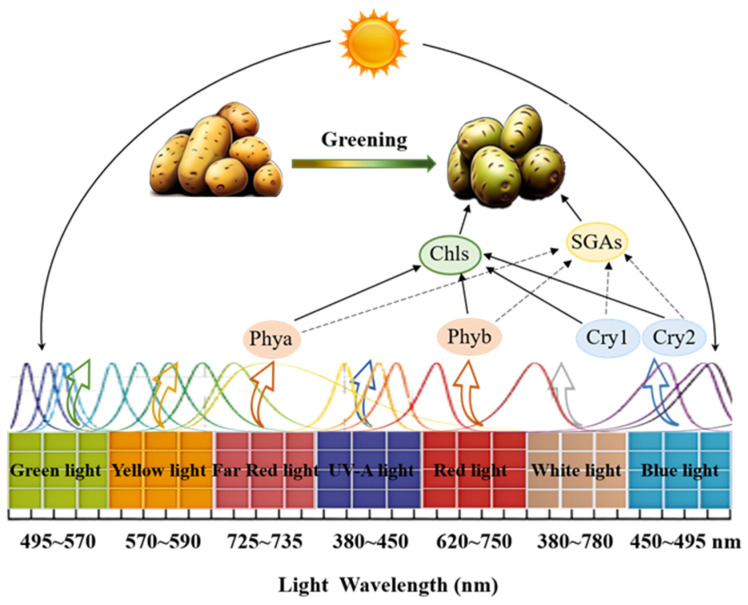
This illustration shows the impact of different light wavelengths on greening in potatoes. Chlorophyll and SGA accumulation increased from green light (left) to blue light (right). Abbreviations: chl, chlorophyll. phya/phyb: phytochromes a/b. Cry1/Cry2: cryptochromes 1/2. 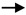 Positive regulation. 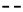 The dotted line needs to be verified by testing.

Moreover, tubers exposed to far-red light showed a significant reduction in chlorophyll and SGA levels, highlighting the crucial role of phytochrome in chlorophyll and SGA accumulation. In summary, the production and accumulation of chlorophyll and SGAs are regulated by gene expression at different stages of the process, and the expression of key genes varies significantly under various light wavelengths (Figure 3). However, at present, technical approaches to mitigating tuber greening through spectral manipulation remain at an exploratory stage and have yet to be systematically studied or developed. So, it is crucial to thoroughly study the molecular mechanisms involved in chlorophyll and SGA production and accumulation in greening potato varieties exposed to different spectra.

### 2.3. Light Intensity

The intensity of light plays a significant role in influencing the greening process in potato tubers. Similarly, Yamaguchi conducted an experiment to assess the effects of different light intensities on potato tuber greening and found that greening was lower under 9.15 μmol/m^2^/s than under 18.29 μmol/m^2^/s. However, greening did not increase further when the light intensity exceeded 18.29 μmol/m^2^/s [31]. That same year, Liljemark studied the impact of varying light intensities (5.10–20.40 μmol/m^2^/s) on chlorophyll and SGA accumulation in potato tubers, and the findings indicated negligible chlorophyll accumulation at 5.10 μmol/m^2^/s and reduced levels at 20.40 μmol/m^2^/s. The range of light intensity was subsequently expanded to 0.85 to 122.40 μmol/m^2^/s, with results indicating that chlorophyll accumulation between 5.10 and 20.40 μmol/m^2^/s was consistent with previous experiments. Chlorophyll formation in tubers is highly responsive to low light conditions, likely involving phytochrome photoreceptors. The SGA levels in Majestic cultivar potato tubers significantly increased under higher light intensities, particularly between 18.43 μmol/m^2^/s and 2675.97 μmol/m^2^/s [32]. Ansitis and Northcote similarly observed significant chlorophyll accumulation in tubers at a light intensity of 25.50 μmol/m^2^/s. This research identifies the optimal light intensity range at which greening no longer increases [8]. Patil’s research later revealed higher chlorophyll formation at 36.58 and 54.88 μmol/m^2^/s light intensities compared to 18.29 and 73.17 μmol/m^2^/s, with the most significant accumulation occurring at 36.58 μmol/m^2^/s. Chlorophyll formation increased with light intensity below 36.58 μmol/m^2^/s, but gradually declined as it approached 1614 lux, suggesting that higher light intensities may degrade existing chlorophyll [35].

**Figure 4:**
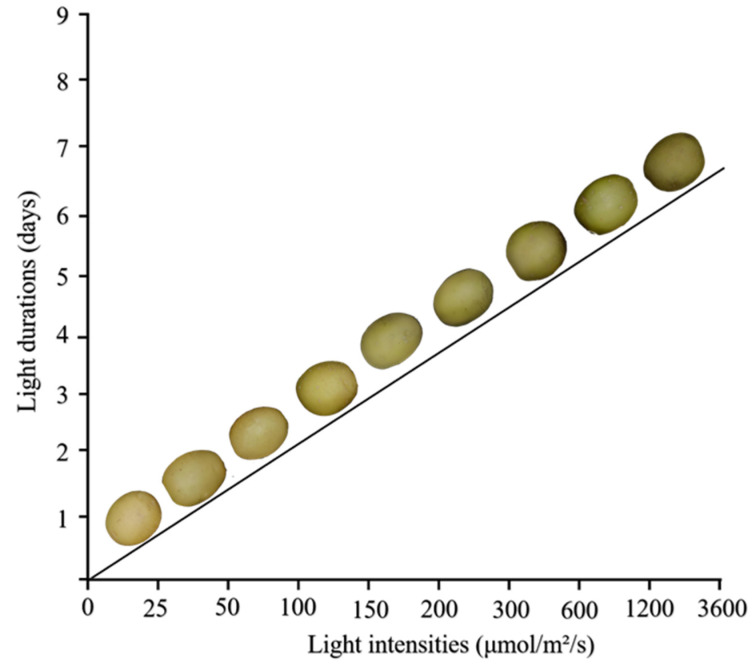
This illustration depicts the effects of varying light durations and intensities on potato greening. The horizontal axis represents light intensity, and the vertical axis represents the duration of light exposure.

Research on retail lighting environments revealed a wide range of light intensities for potatoes, spanning from 2.23–4.47 μmol/m^2^/s to 14.15–34.25 μmol/m^2^/s, with an average range between 0.49275 and 3.55875 μmol/m^2^/s. Additionally, Tanios reported significant variations in brightness levels across different sections of storage shops and display areas, ranging from 2.23 to 34.25 μmol/m^2^/s [34]. However, chlorophyll accumulation remained stable even as light intensity decreased to 2.83 μmol/m^2^/s in Grunenfelder’s study of retail stores [3]. In summary, the risk of potato greening varies across retail environments and is closely related to light intensity. To minimize SGA accumulation, store lighting should be maintained below 18.62 μmol/m^2^/s (Figure 4).

### 2.4. Light Duration

Light duration has a direct impact on the shelf life of tubers and, when combined with other lighting conditions, plays a critical role in greening and SGA synthesis in potato tubers. Larsen’s research revealed an increase in chlorophyll levels in White Rose potato tubers as light exposure duration extended [37]. Folsom observed that after 4 days of fluorescent light exposure, Katahdin yams exhibited pale greening on their skin, which extended to approximately 0.16 cm after 7 days, indicating that longer light exposure enhances greening [38]. In Lutz’s experiments, greening emerged in potato tubers exposed to fluorescent light; the findings indicated that tubers began to show slight greening after 2 h of irradiation, which became noticeable after 3 h, reaching 18.9%. In contrast, intense greening occurred after 7 h of irradiation, with rates reaching 76.9% [47]. Northcote found that chlorophyll accumulation begins after 19 h of light exposure, with chlorophyll a produced more rapidly than chlorophyll b, and significantly increased after three weeks [8]. Martin observed similar results in experiments involving fluorescent light exposure. Chlorophyll accumulation in the tubers of both Rua and Ilam Hardy cultivars increased as the exposure period extended to 5, 10, and 15 days, with the Ilam Hardy cultivar showing intensified greening. The chlorophyll levels in the Rua cultivar rose from 1.445 μg/mL to 3.358 μg/mL, and in the Ilam Hardy strain, chlorophyll levels increased from 1.416 μg/mL to 2.407 μg/mL [39]. Okamoto conducted an experiment exposing KE tubers to white light for 7 days. He observed a gradual increase in chlorophyll levels corresponding to the increase in light exposure duration [16].

When exposed to 100 ft-c of while fluorescent light for 5 days, tubers of Bounty, Kennebec, Norchip, and Red Lasoda were most sensitive to greening and solanine development. LaChipper and Platte tubers were resistant to the effects of light [35]. Percival measured the overall SGA levels in potato tubers of white-skinned (cv Pentland Hawk), pink-skinned (cv Kerrs Pink), and red-skinned (cv Desiree) cultivars after 21 days of daylight exposure. The study found that SGA accumulation in the three cultivars slightly decreased during the first day, then consistently increased, reaching an average of 761 mg/kg FW by day 21 [48]. Mekapogu also observed a consistent rise in tuber SGA levels as time progressed [23]. Similarly, Rymuza studied SGA accumulation in tubers of five potato cultivars over 0, 7, and 14 days of exposure and found a significant increase in SGA content throughout the treatment in all cultivars [49]. Baur harvested 12 potato cultivars irradiated with LED light and observed changes in SGA content in tubers at different intervals (1, 7, and 16 days). The results indicated that SGA content in tubers across all cultivars ranged from 3.0 to 17.1 mg/100 g FW, surpassing the safe threshold of 10 mg/100 g, with notably high SGA accumulation after 16 days of light exposure. In summary, light duration serves as a visual indicator of potato tuber shelf life and is a critical factor in the continuous accumulation of SGAs in tubers [41]. Chlorophyll accumulation and the synthesis of SGAs in potato tubers increased with prolonged light exposure (Figure 4).

## 3. Potato Cultivars to Tolerate Greening

The ability of different potato cultivars to tolerate greening significantly affects the light-induced production of chlorophyll and the accumulation of SGAs. Martin analyzed the chlorophyll accumulation in the tubers of two potato cultivars, Ilam Hardy and Rua, under fluorescent lighting, and discovered that Ilam Hardy tubers had a specific gravity of 1.068 and chlorophyll levels of 0.494 μg/mL, whereas Rua tubers had a specific gravity of 1.085 and chlorophyll levels of 0.358 μg/mL, indicating a significant difference in chlorophyll accumulation between the two cultivars [39]. Reeves assessed the greening response of 144 potato cultivars (16 russet, 29 red, and 99 white) and found that russet tubers accumulated less chlorophyll than white cultivars, with red-skinned cultivars accumulating even lower levels than russets, suggesting that chlorophyll accumulation in potato cultivars may be independent and regulated by distinct genetic mechanisms [42].

Similarly, SGA accumulation in different potato cultivars may occur independently, possibly governed by unique genetic mechanisms. SGA levels in the tubers of Pentland Hawk and Kerrs Pink significantly increased under light exposure, while the Desiree cultivar exhibited lower SGAs accumulation, and Kerrs Pink also showed the fastest SGA accumulation under these conditions. Griffiths also found that the Brodick, Torridon, and Arran Consul cultivars exhibited the fastest average increase in SGA content, tripling the growth rate of the slower-growing Ailsa, Desiree, and Eden cultivars. The GL76B/102 cultivar, which had lower initial SGA levels, showed light sensitivity with a 28 mg/100 g FW increase over 48 h, compared to a 7.7 mg/100 g FW rise in the Ailsa cultivar. In contrast, the Arran Consul cultivar, with elevated initial SGAs levels, showed a significant increase in SGAs under light conditions, while the Desiree tuber SGA levels increased slowly, taking 10 days to exceed 100 mg/100 g FW. The GL76B/102 cultivar showed a 65% reduction in SGA content compared to Desiree. This highlights the importance of considering both the slow SGA accumulation rate under light and the initial SGA levels when selecting cultivars [36]. Percival further revealed that although all varieties showed elevated SGA levels compared to their initial levels after light exposure, these concentrations varied over time and did not accumulate steadily [48].

**Figure 5:**
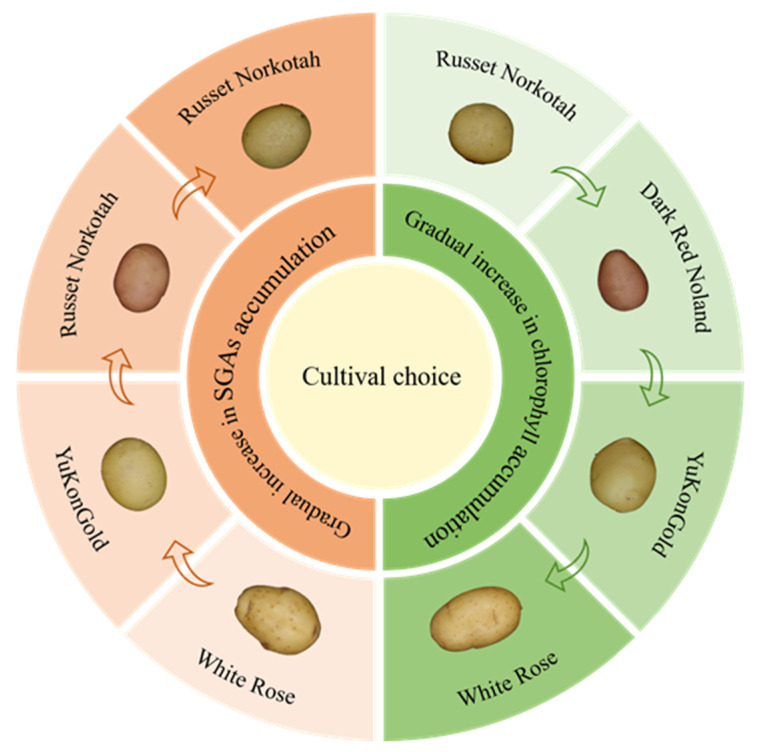
This illustration depicts changes in chlorophyll and SGA levels in various potato varieties during light-induced greening. Green arrows indicate increases in chlorophyll, while orange arrows indicate increases in SGA content.

Different potatoes cultivars exhibited varied responses to light sources induced greening in retail settings. Grunenfelder simulated synthetic lighting in retail shops and compared chlorophyll and SGA concentrations among four potato varieties, White Rose, Yukon Gold, Russet Norkotah, and Dark Red Norland, and found that chlorophyll levels in White Rose and Yukon Gold increased 17-fold and 20-fold, respectively, while Russet Norkotah and Dark Red Norland experienced 5.4-fold and 6-fold increases, respectively. Therefore, chlorophyll levels of the cultivars were ordered from White Rose, Yukon Gold, Dark Red Norland, to Russet Norkotah. SGA levels also varied, with White Rose potato skins ranging from 76 to 138 mg/100 g DW, Yukon Gold periderm from 44 to 110 mg/100 g DW, Dark Red Norland periderm from 38 to 159 mg/100 g DW, and Russet Norkotah periderm exhibiting levels between 37.3 and 98.2 mg/100 g DW. Dark Red Norland, Russet Norkotah, Yukon Gold, and White Rose exhibited the highest total SGA concentrations. Additionally, tuberous potatoes showed a 3.4- to 6.8-fold increase in total SGA levels in the periderm compared to the flesh when heavily greened, with notable variations among different cultivars [26]. Sebastian Baur conducted a similar study in 12 potato cultivars and found SGA concentrations in Albatros, Solo, and Bavatop tubers of 17.1 mg/100 g FW, 14.7 mg/100 g FW, and 13.0 mg/100 g FW, respectively, exceeding the safe limit of 10 mg/100 g FW. Under 1–16 days of light exposure, tuber SGA content increased by 78–461%. The Albatros cultivar exhibited minimal variation in SGA concentration during the trial (12.4 to 17.1 mg/100 g FW), with light exposure not significantly increasing levels, possibly due to its higher baseline SGA levels (Figure 5) (Table 2) [41].

Liu investigated light-responsive phenotypic and metabolic variations in tubers of four potato varieties (Stirling, Huaen No. 2, Innovator, and 11FF35-2) under 0–120 h light/dark cycles, identifying distinct responses in greening and SGA accumulation. “Stirling” and “Huaen No. 2” exhibited pronounced light-induced greening with elevated α-solanine and α-chaconine in the epidermis, whereas “Innovator” and “11FF35-2” showed minimal greening and stable glycoalkaloid levels. Proteomic and transcriptomic analyses revealed a critical functional dissociation between resistance to light-induced greening and resistance to SGA accumulation. Chlorophyll synthesis genes (clustered in Cluster 2) were highly expressed in “Innovator” and “11FF35-2” even under prolonged light, yet these varieties maintained controlled chlorophyll deposition, likely through regulatory mechanisms like feedback inhibition or enhanced catabolism, without compromising biosynthetic capacity. In contrast, SGA biosynthetic genes (Clusters 1 and 6) were selectively upregulated in “Stirling” and “Huaen No. 2”, driving toxin production, while “Innovator” and “11FF35-2” suppressed these pathways independently of chlorophyll metabolism, suggesting transcriptional or enzymatic regulation specific to SGA biosynthesis [50]. So, potato cultivars exhibit genotype-specific light responses: some link greening with toxin biosynthesis, while others dissociate chlorophyll metabolism from SGA accumulation. Understanding this mechanistic divergence is essential for selecting varieties that balance shelf life stability (via greening resistance) and food safety (via SGA accumulation resistance) based on distinct genetic profiles rather than correlated phenotypes.

## 4. Molecular Mechanisms of Light-Induced Biosynthesis of Chlorophyll and SGAs

**Figure 6:**
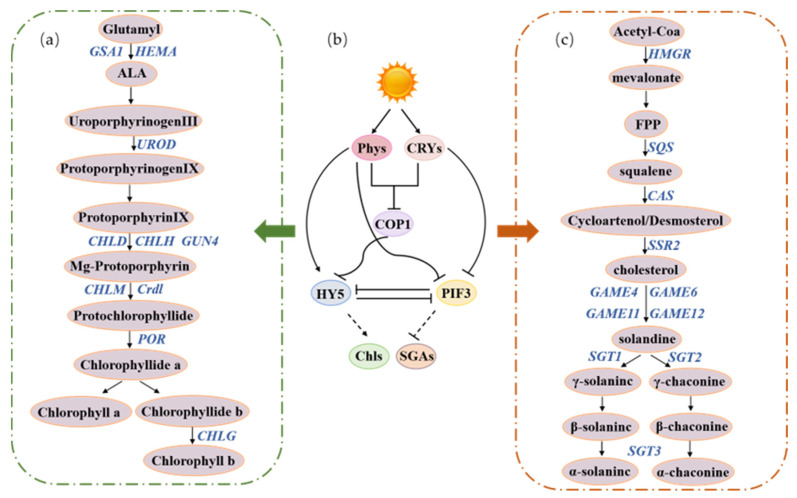
This illustration depicts the pathways and key genes involved in the synthesis of chlorophyll and SGAs during the photoinduced greening of potato tubers. (**a**) Chlorophyll synthesis pathway. (**b**) Molecular mechanism of light-induced chlorophyll and SGA biosynthesis. (**c**) SGA synthesis pathway. 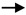 Positive regulation. 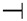 Negative regulation. 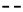 Mechanisms are unclear and need further validation by experiments.

Photoinduced chlorophyll synthesis and SGA accumulation in greening potato tubers involve a series of enzymatic reactions. Early research proved that chlorophyll synthesis begins with Mg-chelatase (MgCh), a StCHLI, StCHLD, and StCHLH complex that uses ATP to insert Mg^2+^ into protoporphyrin IX [51,52]. *StGUN4*, a protein that binds to porphyrin and acts as a positive regulator, facilitates the formation of Mg-protoporphyrin IX (MgPPIX) through MgCh mediation [53]. The enzyme Magnesium protoporphyrin methyltransferase (StCHLM) transforms MgPPIX into Mg-protoporphyrin IX monomethyl ester (MgPME), employing S-adenosyl-methionine (SAM) as the methyl donor [54]. Mg-PROIX monomethylester (oxidized) ring-cyclase (AtCHL27 in Arabidopsis, CrCRD1/CrCTH1 in Chlamydomonas) catalyzes the formation of f 3,8-divinyl protochlorophyllide (DV Pchlide a) and the E ring, shifting the absorption peak to 630 nm, a crucial step in chlorophyll a synthesis [55]. Protochlorophyllide oxidoreductase (StPOR) catalyzes the reduction of DV Pchlide to Chlide at the C17=C18 double bond [56]. Subsequently, NADPH serves as a hydrogen donor alongside catalytic prochlorophyll reduction enzyme-dependent 3,8-divinyl (Chlide) and 8-vinyl reductase (StDVR), facilitating the reduction of the 8-position vinyl group on Chlide’s Bring [57]. Over time, chlorophyll synthase (StCHLG) converts Chlide into chlorophyll a through the use of phytyl pyrophosphate (PhyPP) or geranylgeranyl py-rophosphate (GGPP), resulting in the formation of chlorophyll a [58]. Additionally, chlorophyll a oxygenase (StCAO) takes in Chlide a and facilitates its transformation into Chlide b [59] (Figure 6a).

Previous studies have been clear that the process of synthesizing SGAs encompasses both the pre-cholesterol and post-cholesterol pathways. Within the cholesterol pathway, acetyl coenzyme A engages in the mevalonate (MVA) pathway, resulting in the creation of isoprenyl pyrophosphate (IPP) and dimethylallyl diphosphate (DMAPP). Following this, enzymes including squalene synthase (StSQS), squalene epoxidase (StSQE), and cycloartenol synthase (StCAS) work in unison to aid in the creation of cycloartenol [60]. As cholesterol progresses into the post-cholesterol phase, it experiences various hydroxylation processes [61], driven by cytochrome P450 monooxygenases (CYPs), eventually transforming into 16,22,26-trihydroxycholesterol [50]. Within this group, Glycoalkaloid Metabolism 6 (StGAME6)/StPGA2, StGAME8/StPGA1, and StGAME11/St16DOX play a key role in introducing hydroxyl groups at the C-22, C-26, and C-16 positions, respectively. After oxidation by StGAME4/StPGA3 and StGAME12/StPGA4, an aminotransferase adds a nitrogen atom at the C-26 position, leading to the production of dehydro tomato peptides. Subsequently, in UDP-glycosyltransferases (UGTs), solanidine galactosyltransferase 1 (StSGT1), StSGT2, and StSGT3 contribute to the incorporation of diverse glycosidic units into the glycoside element, leading to structural variances between α-solanine and α-chaconine [62]. When the glycosylation is complete, Dioxygenase for Potato Solanidane synthesis (StDPS) catalyzes the ring arrangement from spirosolanes to solanidane [63]. There are numerous catalytic enzyme genes involved in the SGA biosynthetic pathway, covering 3-hydroxy-3-methylglutaryl-coenzyme A reductase (StHMGR1), keratine synthase (StPSS1, StSQE), C5-desaturase (StC5-SD/StDWF7-L), 16α-hydroxylase (St16DOX), various hydroxylases (StGAME4, StGAME6, StGAME8, StGAME11), transketolase (StGAME12), and glycosyltransferases StSGT1, StSGT2, and StSGT3 [50,62] (Figure 6c).

Plants have different photoreceptors known to regulate chlorophyll and SGA accumulation such as phytochromes (phyA-phyE), cryptochromes (CRY1/CRY2), F-boxes containing lutein binding proteins, UVB-resistance 8 (UVR8), and phototropins (PHOT1/PHOT2) can sense light signals of different wavelengths [64]. Of these, phyA/phyB sensing distant and red light and CRY1/CRY2 sensing blue light play a key role in regulating chlorophyll synthesis [65]. In dark conditions, the phytochrome interaction factor (PIF), a fundamental bHLH transcription factor with a helical loop structure, accumulates and inhibits light morphogenesis. At the same time, cyclic E3 ubiquitin lipase CONSTITUTIVE PHOTO-MORPHOGENIC 1 (StCOP1) forms a complex with phytochrome A (SPA) inhibitors and negatively affects photosynthesis by degrading ELONGATED HYCOTY (StHY5) [66]. StHY5 is a key regulator of light signaling and chloroplast development [67]. Activated phyA/phyB interacts with StPIFs under illumination, triggering StPIFs phosphorylation, ubiquitination, and ultimately degradation by 26S proteasome [68]. On the other hand, StHY5 inhibits StCOP1/SPA activity by directly interacting with or promoting SPA2 degradation [69]. Results showed that mutants containing *StPIF1*, *StPIF3*, *StPIF4*, and *StPIF5* had significantly elevated mRNA levels in the dark for the tetrapyrrole biosynthesis gene (TBS), suggesting that *StPIF4* inhibits chlorophyll synthesis [70,71]. StPIFs inhibit the expression of key TBS genes such as *StHEMA1*, *StCHLH*, and *StGUN4* and promotes the expression of PURA in the dark [72]. StPIFs also synergistically regulates *StCHLH* and *CAB* expression with StCOP1 [73]. Light exposure leads to the degradation of StPIF and the accumulation of positive factors such as StHY5, which binds to the G box in the *StCHLH* and *StPORC* promoters. This process triggers the expression of *StHEMA1* and enhances chlorophyll synthesis [72]. The transcription factor FAR-RED ELONGATED HYPOCOTYL 3 (FHY3) binds the *StHEMB1* and *StHEMA1* gene promoters and regulates chlorophyll synthesis [74]. So, light signal transcription factors regulate chlorophyll synthesis through the modulation of genes involved in the chlorophyll synthesis pathway. However, the mechanism by which light signals regulate chlorophyll synthesis in greening tubers remains unreported.

Previous research also found that key regulators involved in regulating potato SGA synthesis include StHY5, StPIF3, StMYB113, and the AP2/ERF transcription factor [50,75,76]. Research has found that GAME9 is co-expressed with 37 genes involved in SGA biosynthesis, activates the transcription of downstream genes such as DWF5, C5-SD, StGAME4, and StSSR2, binds to the GCC-box, G-box, and GC-rich regions, and synergistically interacts with the jasmonate signaling factor MYC2, enhancing the expression of *HMGR1*, *StGAME7*, and *StGAME17*. Overexpression of *StGAME9* significantly upregulated the expression of several genes in the SGA biosynthesis pathway [77,78]. Liu identified StMYB113 as an active regulator of steroidal glycoalkaloid biosynthesis, which binds to and activates the promoter of related genes [50]. Under light conditions, the light-responsive transcription factor SlHY5 regulates the expression of genes involved in SGA biosynthesis, including *SlGAME1*, *SlGAME4*, and *SlGAME17* in tomato, while SlPIF3 inhibits the expression of these genes [75]. In potato tubers, light signals regulate the biosynthesis of steroidal glycoalkaloids (GAs) through the phytochrome (StPHY) family genes [77]. The five family members (StPHYA/B/B2/C/E) perceive light signals via their conserved N-terminal PAS-GAF-PHY domains, initiating transcriptional regulation mediated by light-responsive elements in the nucleus. Red light specifically activates the photostable receptors StPHYB and StPHYC, whose sustained high expression (peaking at 12 days) shows a strong positive correlation with the contents of α-solanine and α-chaconine. These receptors drive the expression of key genes in both the pre-cholesterol pathway (mevalonate, MVA) and post-cholesterol modification pathways (CYP450 hydroxylation, UGT glycosylation), leading to SGA enrichment in the outer 1 mm of the tuber periderm. This process exhibits tissue specificity (highest PHY expression in flowers) and cultivar diversity: some low-greening cultivars uncouple chlorophyll metabolism from toxin synthesis by suppressing StPHYB/C-mediated activation of SGA biosynthetic pathways. The study reveals a core regulatory pathway of “red light-StPHYB/C-SGAs synthase genes”, providing critical targets for deciphering the molecular mechanisms of light-induced toxin accumulation and breeding SGA-resistant potato cultivars (Figure 6b) [78]. Although the molecular mechanisms underlying light-regulated chlorophyll and SGA biosynthesis have been studied in various plant species, their specific regulatory pathways in greening tubers remain unclear, and several important questions remain to be explored. For instance, are there more complex synergistic or antagonistic relationships among different photoreceptors? Additionally, are there undiscovered regulatory factors that act either upstream or downstream of the key regulators, StPIF3 and StHY5? Does a co-regulatory mechanism exist between chlorophyll synthesis and SGA accumulation in the photoinduced greening of potato tubers?

## 5. Conclusions and Prospects

The greening of potato tubers under light exposure conditions leads to the accumulation of high concentrations of secondary metabolites, specifically SGAs, which significantly reduce tuber quality and increase food safety risks. These further research strategies aimed at reducing tuber greening, extending shelf life, and improving the overall safety of potato products should focus on the following aspects:

(1) The advancement of LED technology offers the potential to optimize ambient lighting in retail displays [79]. However, at present, technical approaches to mitigating tuber greening through spectral manipulation remain at an exploratory stage and have yet to be systematically studied or developed. Future research requires precise control of lighting conditions—such as light source, wavelength, intensity, and duration—to effectively manage tuber greening, reduce the negative impact on tuber quality throughout its shelf life, improve visual appeal, and safeguard consumer health.

(2) Nearly seven decades of research have explored how light intensity, duration, and wavelength contribute to tuber greening [8,24,34,80]. Despite this extensive research, the regulatory mechanisms underlying light-induced chlorophyll production and SGA accumulation remain unclear. Further investigation is required to elucidate the genetic basis of light-induced tuber greening. Such research will help identify relevant genes and regulatory elements, laying the groundwork for the development of greening-resistant potato cultivars.

## Figures and Tables

**Table 1:** Main research reviews of the potato greening response under different light conditions.

	Influencing Factors	Crucial Role	References
Light conditions	Light sources	The effects of various light sources on greening rate and chlorophyll synthesis in greening tubers.	[6,24,25,26]
		The accumulation of SGAs of greening tubers when exposed to various light sources.	[24,27,28,29,30]
	Light wavelengths	The effects of various light wavelengths on greening rate and chlorophyll synthesis in greening tubers.	[16,31,32,33,34]
		The accumulation of SGAs of greening tubers when exposed to various light wavelengths.	[14,16,31,32,33,34]
	Light intensity	The effects of various light intensity on greening rate and chlorophyll synthesis in greening tubers.	[8,31,32,35]
		The accumulation of SGAs of greening tubers when exposed to various light intensity.	[3,26,32,34,36]
	Light durations	The effects of various light durations on greening rate and chlorophyll synthesis in greening tubers.	[14,37,38,39]
		The accumulation of SGAs of greening tubers when exposed to various light durations.	[33,39,40,41]
Cultivars	Pigment accumulation	Accumulation of pigmentation in the tuber periderm reduced greening change.	[3,26,41,42]
	Suberin of tuber periderm	Cultivars susceptible to greening are strongly associated with higher suberin deposition in the tuber periderm.	[26]

**Table 2:** Comparison table of SGA accumulation in potato tubers of different varieties.

Cultivars	Light Conditions	Treatment Duration	SGA Concentration (mg/100 g FW or DW)	Characteristics	References
Pentland Hawk	Fluorescent light/natural light	21 days	25.41 (fluorescent); 761 mg/kg FW (natural light)	White-skinned variety	[29,48]
Kerrs Pink	Fluorescent light/sodium light	21 days	22.49 (fluorescent)	Pink-skinned variety	[29,36]
Desiree	Fluorescent light/sodium light	10–21 days	Low accumulation (exceeding 10 mg/100 g FW after 10 days)	Red-skinned variety	[24,36]
White Rose	Fluorescent light/retail LED	10 days	76–138 (periderm, DW)	Extremely sensitive to greening	[26]
Yukon Gold	Ceramic metal halide light	10 days	44–110 (periderm, DW)	Yellow-skinned variety	[26]
Russet Norkotah	Fiber optic/halogen light	10 days	37.3–98.2 (periderm, DW)	Russet-skinned variety	[26]
Dark Red Norland	Fluorescent light	10 days	38–159 (periderm, DW)	Red-skinned variety	[26]
Albatros	LED light	1–16 days	12.4–17.1 (stable, FW)	Variety with high baseline SGAs	[41]
Solo	LED light	16 days	14.7 (peak, FW)	Moderately sensitive	[41]
Brodick	Natural light	48 h	3× SGAs increase compared to Ailsa	Variety with high SGA accumulation rate	[36]
GL76B/102	Fluorescent light	48 h	28 mg/100 g FW (65% increase vs. Desiree)	Low initial SGAs variety	[36]

## Data Availability

No new data were created or analyzed in this study. Data sharing is not applicable to this article.

## References

[1] Birch P.R.J., Bryan G., Fenton B., Gilroy E.M., Hein I., Jones J.T., Prashar A., Taylor M.A., Torrance L., Toth I.K. (2012). Crops that feed the world 8: Potato: Are the trends of increased global production sustainable?. Food Secur..

[2] Faostat. https://www.fao.org/faostat.

[3] Grunenfelder L. (2005). Physiological Studies of Light-Induced Greening in Fresh Market Potatoes. Master’s Thesis.

[4] Townsend J.N. (2021). Potato Field Greening and Response to Potassium Fertilization in the Columbia Basin. Ph.D. Thesis.

[5] French-Brooks J. (2012). Reducing Supply Chain and Consumer Potato Waste.

[6] Bamberg J., Moehninsi, Navarre R., Suriano J. (2015). Variation for tuber greening in the diploid wild potato solanum microdontum. Am. J. Potato Res..

[7] Muraja-Fras J., Krsnik-Rasol M., Wrischer M. (1994). Plastid transformation in greening potato tuber tissue. J. Plant Physiol..

[8] Anstis P., Northcote D. (1973). Development of chloroplasts from amyloplasts in potato tuber discs. New Phytol..

[9] Zhu Y.S., Merkle-Lehman D.L., Kung S.D. (1984). Light-induced transformation of amyloplasts into chloroplasts in potato tubers. Plant Physiol..

[10] Mochizuki N., Tanaka R., Grimm B., Masuda T., Moulin M., Smith A.G., Tanaka A., Terry M.J. (2010). The cell biology of tetrapyrroles: A life and death struggle. Trends Plant Sci..

[11] Pavlista A.D. (2001). Green Potatoes: The Problem and the Solution.

[12] Dhalsamant K., Singh C.B., Lankapalli R. (2022). A review on greening and glycoalkaloids in potato tubers: Potential solutions. J. Agric. Food Chem..

[13] Dao L., Friedman M. (1994). Chlorophyll, chlorogenic acid, glycoalkaloid, and protease inhibitor content of fresh and green potatoes. J. Agric. Food Chem..

[14] Edwards E.J., Cobb A.H. (1997). Effect of temperature on glycoalkaloid and chlorophyll accumulation in potatoes (*Solanum tuberosum* L. cv. King Edward) stored at low photon flux density, including preliminary modeling using an artificial neural network. J. Agric. Food Chem..

[15] Edwards E.J., Saint R.E., Cobb A.H. (1998). Is there a link between greening and light-enhanced glycoalkaloid accumulation in potato (*Solanum tuberosum* L.) tubers?. J. Sci. Food Agric..

[16] Okamoto H., Ducreux L.J.M., Allwood J.W., Hedley P.E., Wright A., Gururajan V., Terry M.J., Taylor M.A. (2020). Light regulation of chlorophyll and glycoalkaloid biosynthesis during tuber greening of potato *S. tuberosum*. Front. Plant Sci..

[17] Maga J.A. (1994). Glycoalkaloids in solanaceae. Food Rev. Int..

[18] Sinden S., Deahl K., Aulenbach B. (1976). Effect of glycoalkaloids and phenolics on potato flavor. J. Food Sci..

[19] Takagi K., Toyoda M., Fujiyama Y., Saito Y. (1990). Effect of cooking on the contents of α-chaconine and α-solanine in potatoes. J. Food Hyg. Soc. Jpn..

[20] Morris S.C., Lee T.H. (1984). The toxicity and teratogenicity of solanaceae glycoalkaloids, particularly those of the potato (*Solanum tuberosum* L.): A review. Food Aust..

[21] Schrenk D., Bignami M., Bodin L., Chipman J.K., del Mazo J., Hogstrand C., Hoogenboom L., Leblanc J.C., Nebbia C.S., Nielsen E. (2020). Risk assessment of glycoalkaloids in feed and food, in particular in potatoes and potato-derived products. EFSA J..

[22] Mensinga T.T., Sips A.J.A.M., Rompelberg C.J.M., van Twillert K., Meulenbelt J., van den Top H.J., van Egmond H.P. (2005). Potato glycoalkaloids and adverse effects in humans: An ascending dose study. Regul. Toxicol. Pharm..

[23] Mekapogu M., Sohn H.-B., Kim S.-J., Lee Y.-Y., Park H.-M., Jin Y.-I., Hong S.-Y., Suh J.-T., Kweon K., Jeong J.-C. (2016). Effect of light quality on the expression of glycoalkaloid biosynthetic genes contributing to steroidal glycoalkaloid accumulation in potato. Am. J. Potato Res..

[24] Percival G. (1999). The influence of light upon glycoalkaloid and chlorophyll accumulation in potato tubers (*Solanum tuberosum* L.). Plant Sci..

[25] Olsen N.L., Brandt T., Price W.J. (2017). The impact of retail light source on greening of russet burbank potato tubers. Am. J. Potato Res..

[26] Grunenfelder L.A., Knowles L.O., Hiller L.K., Knowles N.R. (2006). Glycoalkaloid development during greening of fresh market potatoes (*Solanum tuberosum* L.). J. Agric. Food Chem..

[27] Conner H.W. (1937). Effect of light on solanine synthesis in the potato tuber. Plant Physiol..

[28] Machado R.M.D., Toledo M.C.F., Garcia L.C. (2007). Effect of light and temperature on the formation of glycoalkaloids in potato tubers. Food Control..

[29] Percival G., Harrison J., Dixon G. (1993). The influence of temperature on light enhanced glycoalkaloid synthesis in potato. Ann. Appl. Biol..

[30] Percival G., Dixon G., Sword A. (1994). Glycoalkaloid concentration of potato tubers following continuous illumination. J. Sci. Food Agric..

[31] Yamaguchi M., Hughes D., Howard F. (1960). Effect of color and intensity of fluorescent lights and application of chemicals and waxes on chlorophyll development of White Rose potatoes. Am. Potato J..

[32] Liljemark A., Widoff E. (1960). Greening and solanine development of white potatoes in various types of consumer packages. Am. J. Potato Res..

[33] Petermann J.B., Morris S.C. (1985). The spectral responses of chlorophyll and glycoalkaloid synthesis in potato tubers (*Solanum tuberosum*). Plant Sci..

[34] Tanios S., Eyles A., Corkrey R., Tegg R.S., Thangavel T., Wilson C.R. (2020). Quantifying risk factors associated with light-induced potato tuber greening in retail stores. PLoS ONE.

[35] Patil B., Salunkhe O., Singh B. (1971). Metabolism of solanine and chlorophyll in potato tubers as affected by light and specific chemicals. J. Food Sci..

[36] Griffiths D., Dale M., Bain H. (1994). The effect of cultivar, maturity and storage on photo-induced changes in the total glycoalkaloid and chlorophyll contents of potatoes (*Solanum tuberosum* L.). Plant Sci..

[37] Larsen E. (1949). Investigations on Cause and Prevention of Greening of Potato Tubers.

[38] Folsom D. (1947). Permanence of greening of potato tubers. Am. Potato J..

[39] Martin S.K., Sheppard R.L. (1983). Effect of different packaging materials and light exposure times on chlorophyll concentration in 2 cultivars of potato. N. Z. J. Agric. Res..

[40] Şengül M., Keleş F., Keleş M.S. (2004). The effect of storage conditions (temperature, light, time) and variety on the glycoalkaloid content of potato tubers and sprouts. Food Control..

[41] Baur S., Bellé N., Hausladen H., Wurzer S., Brehm L., Stark T.D., Hücklhoven R., Hofmann T., Dawid C. (2022). Quantitation of toxic steroidal glycoalkaloids and newly iIdentified saponins in post-harvest light-stressed potato (*Solanum tuberosum* L.) Varieties. J. Agric. Food Chem..

[42] Reeves A.F. (1988). Varietal differences in potato tuber greening. Am. Potato J..

[43] Salunkhe D., Wu M., Jadhav S. (1972). Effects of light and temperature on the formation of solanine in potato slices. J. Food Sci..

[44] Heyes D.J., Hunter C.N. (2005). Making light work of enzyme catalysis: Protochlorophyllide oxidoreductase. Trends Biochem. Sci..

[45] Haverkort A., Linnemann A., Struik P., Wiskerke J. (2023). On processing potato 3: Survey of performances, productivity and losses in the supply chain. Potato Res..

[46] Mancinelli A.L., Rabino I. (1978). The “high irradiance responses” of plant photomorphogenesis. Bot. Rev..

[47] Lutz J., Findlen H., Ramsey G. (1951). Quality of red river valley potatoes in various types of consumer packages. Am. Potato J..

[48] Percival G., Dixon G.R., Sword A. (1996). Glycoalkaloid concentration of potato tubers following exposure to daylight. J. Sci. Food Agric..

[49] Rymuza K., Gugała M., Zarzecka K., Sikorska A., Findura P., Malaga-Toboła U., Kapela K., Radzka E. (2020). The effect of light exposures on the content of hharmful substances in edible potato tuber. Agriculture.

[50] Liu S., Cheng Y., Zhao X., Wang E., Liu T., Zhang H., Liu T., Botao S. (2024). The transcription factor StMYB113 regulates light-induced greening by modulating steroidal glycoalkaloid biosynthesis in potatoes (*Solanum tuberosum* L.). Hortic. Adv..

[51] Yaronskaya E., Grimm B. (2006). The pathway from 5-aminolevulinic acid to protochlorophyllide and protoheme. Chlorophylls Bacteriochlorophylls Biochem. Biophys. Funct. Appl..

[52] Tanaka R., Tanaka A. (2007). Tetrapyrrole biosynthesis in higher plants. Annu. Rev. Plant Biol..

[53] Richter A.S., Nägele T., Grimm B., Kaufmann K., Schroda M., Leister D., Kleine T. (2023). Retrograde signaling in plants: A critical review focusing on the GUN pathway and beyond. Plant Commun..

[54] Shepherd M., Reid J.D., Hunter C.N. (2003). Purification and kinetic characterization of the magnesium protoporphyrin IX methyltransferase from Synechocystis PCC6803. Biochem. J..

[55] Tottey S., Block M.A., Allen M., Westergren T., Albrieux C., Scheller H.V., Merchant S., Jensen P.E. (2003). Arabidopsis CHL27, located in both envelope and thylakoid membranes, is required for the synthesis of protochlorophyllide. Proc. Natl. Acad. Sci. USA.

[56] Apel K., Santel H.J., Redlinger T.E., Falk H. (2005). The protochlorophyllide holochrome of barley (*Hordeum vulgare* L.). Eur. J. Biochem..

[57] Nagata N., Tanaka R., Satoh S., Tanaka A. (2005). Identification of a vinyl reductase gene for chlorophyll synthesis in arabidopsis thaliana and implications for the evolution of prochlorococcus species. Plant Cell.

[58] Keller Y., Bouvier F., d’Harlingue A., Camara B. (2001). Metabolic compartmentation of plastid prenyllipid biosynthesis. Eur. J. Biochem..

[59] Oster U., Tanaka R., Tanaka A., Rüdiger W. (2001). Cloning and functional expression of the gene encoding the key enzyme for chlorophyll b biosynthesis (CAO) from arabidopsis thaliana. Plant J..

[60] Akiyama R., Umemoto N., Mizutani M. (2023). Recent advances in steroidal glycoalkaloid biosynthesis in the genus solanum. Plant Biotechnol..

[61] Grzech D., Smit S.J., Alam R.M., Boccia M., Nakamura Y., Hong B., Barbole R., Heinicke S., Kunert M., Seibt W. (2024). Incorporation of nitrogen in antinutritional solanum alkaloid biosynthesis. Nat. Chem. Biol..

[62] McCue K.F., Breksa A., Vilches A., Belknap W.R. (2017). Modification of potato steroidal glycoalkaloids with silencing RNA constructs. Am. J. Potato Res..

[63] Akiyama R., Watanabe B., Nakayasu M., Lee H.J., Kato J., Umemoto N., Muranaka T., Saito K., Sugimoto Y., Mizutani M. (2021). The biosynthetic pathway of potato solanidanes diverged from that of spirosolanes due to evolution of a dioxygenase. Nat. Commun..

[64] Paik I., Huq E. (2019). Plant photoreceptors: Multi-functional sensory proteins and their signaling networks. Semin. Cell Dev. Biol..

[65] Sharma S., Sanyal S.K., Sushmita K., Chauhan M., Sharma A., Anirudhan G., Veetil S.K., Kateriya S. (2021). Modulation of phototropin signalosome with artificial illumination holds great potential in the development of climate-smart crops. Curr. Genom..

[66] Pham V.N., Kathare P.K., Huq E. (2018). Phytochromes and phytochrome interacting factors. Plant Physiol..

[67] Zhang T., Zhang R., Zeng X.Y., Lee S., Ye L.H., Tian S.L., Zhang Y.J., Busch W., Zhou W.B., Zhu X.G. (2024). GLK transcription factors accompany ELONGATED HYPOCOTYL5 to orchestrate light-induced seedling development in Arabidopsis. Plant Phys..

[68] Paik I., Chen F., Ngoc Pham V., Zhu L., Kim J., Huq E. (2019). A phyB-PIF1-SPA1 kinase regulatory complex promotes photomorphogenesis inArabidopsis. Nat. Commun..

[69] Lu X.-D., Zhou C.-M., Xu P.-B., Luo Q., Lian H.-L., Yang H.-Q. (2015). Red-light-dependent interaction of phyB with SPA1 promotes COP1–SPA1 dissociation and photomorphogenic development in arabidopsis. Mol. Plant.

[70] Leivar P., Tepperman J.M., Monte E., Calderon R.H., Liu T.L., Quail P.H. (2009). Definition of early transcriptional circuitry involved in light-induced reversal of PIF-imposed repression of photomorphogenesis in young Arabidopsis seedlings. Plant Cell.

[71] Shin J., Kim K., Kang H., Zulfugarov I.S., Bae G., Lee C.-H., Lee D., Choi G. (2009). Phytochromes promote seedling light responses by inhibiting four negatively-acting phytochrome-interacting factors. Proc. Natl. Acad. Sci. USA.

[72] Toledo-Ortiz G., Johansson H., Lee K.P., Bou-Torrent J., Stewart K., Steel G., Rodríguez-Concepción M., Halliday K.J. (2014). The HY5-PIF regulatory module coordinates light and temperature control of photosynthetic gene transcription. PLoS Genet..

[73] Feng C.-M., Qiu Y., Van Buskirk E.K., Yang E.J., Chen M. (2014). Light-regulated gene repositioning in arabidopsis. Nat. Commun..

[74] Wang W., Tang W., Ma T., Niu D., Jin J.B., Wang H., Lin R. (2015). A pair of light signaling factors FHY3 and FAR1 regulates plant immunity by modulating chlorophyll biosynthesis. J. Integr. Plant Biol..

[75] Swinnen G., De Meyer M., Pollier J., Molina-Hidalgo F.J., Ceulemans E., Venegas-Molina J., De Milde L., Fernández-Calvo P., Ron M., Pauwels L. (2022). The basic helix–loop–helix transcription factors MYC1 and MYC2 have a dual role in the regulation of constitutive and stress-inducible specialized metabolism in tomato. New Phytol..

[76] Yu G., Li C., Zhang L., Zhu G., Munir S., Shi C., Zhang H., Ai G., Gao S., Zhang Y. (2020). An allelic variant of GAME9 determines its binding capacity with the GAME17 promoter in the regulation of steroidal glycoalkaloid biosynthesis in tomato. J. Exp. Bot..

[77] Mathews S. (2010). Evolutionary Studies Illuminate the Structural-Functional Model of Plant Phytochromes. Plant Cell.

[78] Zhang X., Jiang H., Liu W., Wang Y., Zeng F. (2023). Effect of red light on the expression of the phytochrome gene family and the accumulation of glycoside alkaloids in potatoes. Foods.

[79] Ouzounis T., Rosenqvist E., Ottosen C.-O. (2015). Spectral effects of artificial light on plant physiology and secondary metabolism: A review. HortScience.

[80] Virgin H.I., Sundqvist C. (1992). Pigment formation in potato tubers (*Solanum tuberosum* L.) exposed to light followed by darkness. Physiol. Plant.

